# A scoping review of evidence-based guidance and guidelines published by general practice professional organizations

**DOI:** 10.1093/fampra/cmad015

**Published:** 2023-02-22

**Authors:** Emer O’Brien, Seamus Duffy, Velma Harkins, Susan M Smith, Noirin O’Herlihy, Aisling Walsh, Barbara Clyne, Emma Wallace

**Affiliations:** Department of General Practice, RCSI University of Medicine and Health Sciences, Dublin, Ireland; Department of General Practice, RCSI University of Medicine and Health Sciences, Dublin, Ireland; Irish College of General Practitioners, Dublin, Ireland; Department of General Practice, RCSI University of Medicine and Health Sciences, Dublin, Ireland; Department of Public Health and Primary Care, Trinity College Dublin, DublinIreland; Irish College of General Practitioners, Dublin, Ireland; Department of Public Health and Epidemiology, RCSI University of Medicine and Health Sciences, Dublin, Ireland; Department of General Practice, RCSI University of Medicine and Health Sciences, Dublin, Ireland; Department of General Practice, RCSI University of Medicine and Health Sciences, Dublin, Ireland; Department of General Practice, University College Cork, Cork, Ireland

**Keywords:** clinical practice guidelines, evidence-based practice, family practitioner, general practice, primary healthcare

## Abstract

**Background:**

General practitioners (GPs) need robust, up-to-date evidence to deliver high-quality patient care. There is limited literature regarding the role of international GP professional organizations in developing and publishing clinical guidelines to support GPs clinical decision making.

**Objective:**

To identify evidence-based guidance and clinical guidelines produced by GP professional organizations and summarize their content, structure, and methods of development and dissemination.

**Methods:**

Scoping review of GP professional organizations following Joanna Briggs Institute guidance. Four databases were searched and a grey literature search was conducted. Studies were included if they were: (i) evidence-based guidance documents or clinical guidelines produced de novo by a national GP professional organization, (ii) developed to support GPs clinical care, and (iii) published in the last 10 years. GP professional organizations were contacted to provide supplementary information. A narrative synthesis was performed.

**Results:**

Six GP professional organizations and 60 guidelines were included. The most common de novo guideline topics were mental health, cardiovascular disease, neurology, pregnancy and women’s health and preventive care. All guidelines were developed using a standard evidence-synthesis method. All included documents were disseminated through downloadable pdfs and peer review publications. GP professional organizations indicated that they generally collaborate with or endorse guidelines developed by national or international guideline producing bodies.

**Conclusion:**

The findings of this scoping review provide an overview of de novo guideline development by GP professional organizations and can support collaboration between GP organizations worldwide thus reducing duplication of effort, facilitating reproducibility, and identifying areas of standardization.

**Protocol registration:**

Open Science Framework: https://doi.org/10.17605/OSF.IO/JXQ26.

**Key messages**

- GP professional organizations produce de novo clinical guidelines.
- Mental health is the most common de novo guideline category.
- Dissemination occurs via downloadable pdfs and peer review publication.
- GP professional organizations also collaborate on and endorse other guidelines.

## Introduction

General practitioners (GPs) practice medicine in the setting of the community and the family,^1^ and are responsible for providing comprehensive and ongoing care to every individual seeking medical support irrespective of their illness, sex, or age.^2^ Internationally there are variations in healthcare structures and the cultural settings that GPs practice in, but there are also similar components. For example in many countries GPs act as gatekeepers, facilitating access to hospital and speciality care and certain diagnostic tests.^3^

GPs endeavour to use a patient centred approach to achieve shared decision making, through the integration of clinical evidence, clinical judgement, and patient priorities.^4,5^ In this setting, accessible, succinct, evidence-based guidance is required by GPs to support patient care.^6–10^ Clinical practice guidelines, being systematically developed statements, based on a comprehensive evaluation of evidence, aim to address this need and support practitioners’ to make healthcare decisions.^11^ However, a review of 45 UK clinical practice guidelines reported a significant number of guideline recommendations were based on studies with little relevance to primary care.^12^ With up to 80% of GP consultations involving the management of patients with multiple chronic medical conditions, GPs require adequate decision support to deliver such complex care in the primary care setting.^13–16^ High workload and time pressure are significant barriers to utilization of clinical guidelines,^17^ however, despite these barriers, GPs are more likely to use guidelines that involved GP contributors during the development stage and where the evidence is applicable to primary care.^18^

Availability of resources and national guideline development agencies influence how GP professional organizations develop and disseminate clinical guidelines.^19^ National guideline agencies may approach GP organizations to endorse their guidelines.^20^ GP organizations may also adapt or adopt existing national and/or international guidelines and disseminate the findings to their members in the context of primary care.^21–23^ Some GP organizations play a central role in developing de novo guidelines for GPs. For example, the Dutch College of GPs/Nederlands Huisartsen Genootschap (NHG) develop clinical guidelines which cover a range of primary care presentations.^24^ Assimilating how GP professional organizations develop and disseminate clinical guidelines for their members, would facilitate collaboration between GP organizations thus reducing duplication of effort and promoting a standardization of processes to support GPs in their clinical decision making.^25^

The aim of this scoping review was to identify what evidence-based guidance is published by GP professional organizations internationally to support GPs in their clinical decision making. The objectives were: (i) to identify the topics covered, (ii) to review the methods used to develop evidence-based guidance and/or clinical guidelines and how these guidance documents are structured, and (iii) to explore how evidence is disseminated to GPs.

## Methods

This scoping review was preregistered on Open Science Framework and a study protocol has been published.^25^

Given that many GP guidelines may not be published as peer reviewed publications and the evident heterogeneity of nomenclature (e.g. guides versus guidance versus clinical guidelines), a combination of a bibliographic database search, grey literature search, and GP organization author contacts was conducted.

### Scoping review study design

The scoping review was conducted in accordance with JBI methodology for scoping reviews^26–28^ and is reported according to the Preferred Reporting Items for Systematic Reviews and Meta-Analysis extension for scoping reviews (PRISMA-ScR).^29^ A scoping review was selected as it is designed to address a broad research question by mapping a body of literature in that area.^30,31^ The research team comprised researchers and knowledge users (GPs and Irish College of General Practitioners) and through regular meetings identified the specific research question, designed the search strategy, and synthesized the evidence.

#### Eligibility

Articles were included where they were an evidence-based guidance document or clinical guideline (henceforth “guideline”) produced by a national GP professional organization, either de novo (new and updated versions) or through adaptation (Table 1). These guidelines had to support GPs clinical decision making and patient clinical care and be published in the last 10 years for currency. For the purposes of this scoping review “published” refers to guidelines that are made freely available by GP professional organizations on their website or through peer reviewed publication. It does not include guidelines that are collaborations with or endorsed by other, non GP organizations. No language restrictions were applied. English versions were sought on the organizations websites or translated via Google translate.

**Table 1.** Summary of the eligibility criteria for the review.

Inclusion criteria	Excluded
National GP professional organization	Regional organizations
Evidence based guidance or guideline development (i) Explicit method of development (ii) Includes literature review (iii) Reviewed by committee or experts (iv) Recommendation formation not necessary once distinction made between guidance and guideline	Commentary Position statement
Published by GP professional organizations (i) Produced de novo or by adaptation (ii) Open access on GP professional organization website and/or (iii) Peer review publication	Collaborations with other non GP specialties or other guideline producing bodies Endorsements by other national or international guideline producing bodies GP organizations with members only access
Published in the last 10 years	Publications >10 years old
All languages	
Patient clinical care	Governance document Policy document
World Organization of National Colleges, Academies and Academic Associations of General Practitioners/Family Physicians (WONCA) definition of GP^2^	

#### Search strategy

The search strategy (Appendix 1) was developed in consultation with an information specialist (PM), and applied to 4 bibliographic databases (Medline, Embase, Cochrane Library, and Scopus). The search was conducted on 12 April 2021.

### Grey literature search

In addition to NHS evidence and Guideline central, websites of included organizations were searched for clarification regarding the production method of de novo guidelines. This search was not exhaustive, e.g. if additional de novo guidelines were discovered from the organization websites these were not charted as per the database search.

#### Screening and data extraction

Two reviewers (SD and EOB) screened titles for eligibility, a third reviewer (EW) resolved conflicts. One reviewer (EOB) completed the data charting, with 20% checked by the second reviewer (SD).

#### Quality appraisal and analysis

Consistent with established scoping review methodology,^26^ we did not appraise the risk of bias of included studies, nor did we summarize the data quantitatively (meta-analysis). Findings were synthesized narratively with descriptive statistics (e.g. frequencies and percentages).

GP professional organizations were contacted to provide supplementary information. A total of 39 organizations were contacted through professional links via the Irish College of General Practitioners (*n* = 6) and links with the European Society for Quality and Safety in Practice (*n* = 33). GP professional organizations were invited to provide additional information regarding their published guidelines (see Appendix 2) which was based on the inclusion criteria for the review (Table 1). Responses were collected using Survey Monkey, transferred to Excel, and data analysed using descriptive statistics. The purpose was to clarify methods of guideline production and publication within GP organizations included in the scoping review and identify any other relevant publications.

## Results

### Search results

**Fig. 1.**
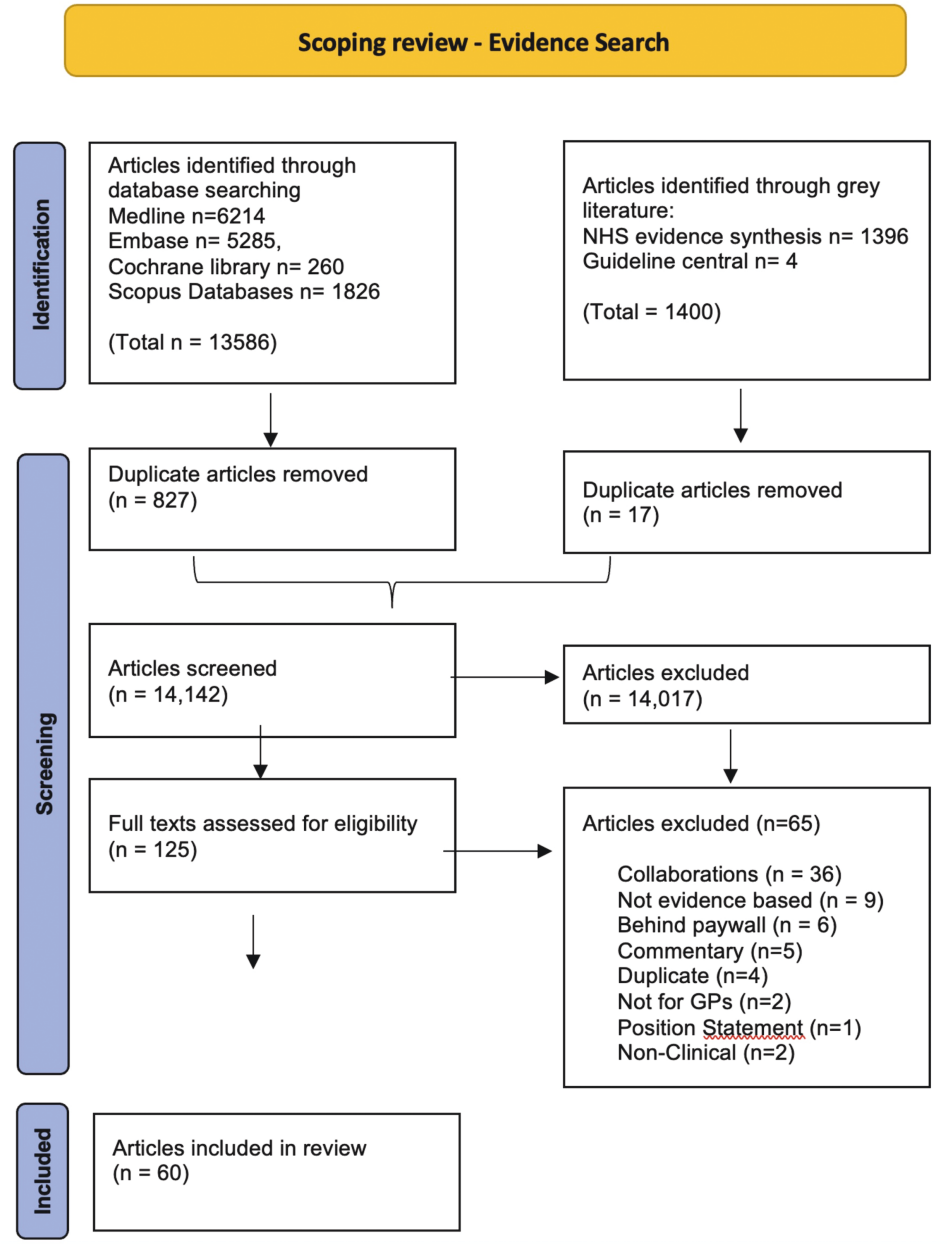
PRISMA flow diagram of articles retrieved for scoping review on evidence-based guidance documents or clinical guidelines produced by general practice professional organizations, 2010–2021.

A total of 14,142 titles and abstracts were identified after duplicates removed. Of these, 125 articles full texts were assessed for eligibility with 60 full texts guidelines being included (Appendix 3 and Fig. 1) from 6 organizations. Findings from the websites of included organizations revealed that additional de novo guidelines not retrieved from the database search were also available. The 60 identified guidelines from the database search are therefore a subset of de novo guidelines from the included organizations.

Of the 39 GP professional organizations contacted to provide supplementary information, 13 responded, representing input from 12 unique GP professional organizations (Appendix 4). Four of these 12 organizations met the inclusion criteria; being national organizations and producing evidence-based de novo guidelines. Eight organizations were excluded based on production method, members only access or being a regional organization (Table 2). Guideline topics covered in the questionnaire included all methods of production. These findings were documented for the purposes of comparison and context, although they were outside the inclusion criteria of de novo production. Of the 6 organizations identified in the database search, 4 responded to the contact for supplementary information and 2 did not.

**Table 2.** Summary of excluded organizations from those contacted.

GP professional organization	Reason for exclusion
Swedish Association of General Practice	Produce guidelines with other specialities
Royal College of General Practitioners	Produce guidelines in association with other national bodies
Hong Kong College of Family Physicians	Do not produce de novo guidelines & regional
Austrian Society of General and Family Physicians	Do not produce de novo guidelines
Greek Association of General Practitioners	Do not produce de novo guidelines & regional
Serbian Medical Society	Do not produce de novo guidelines
Irish College of General Practitioners	Members only access to guidelines
Slovenian Family Medicine Association	Members only access to guidelines

### Characteristics of included guidelines

**Fig. 2.**
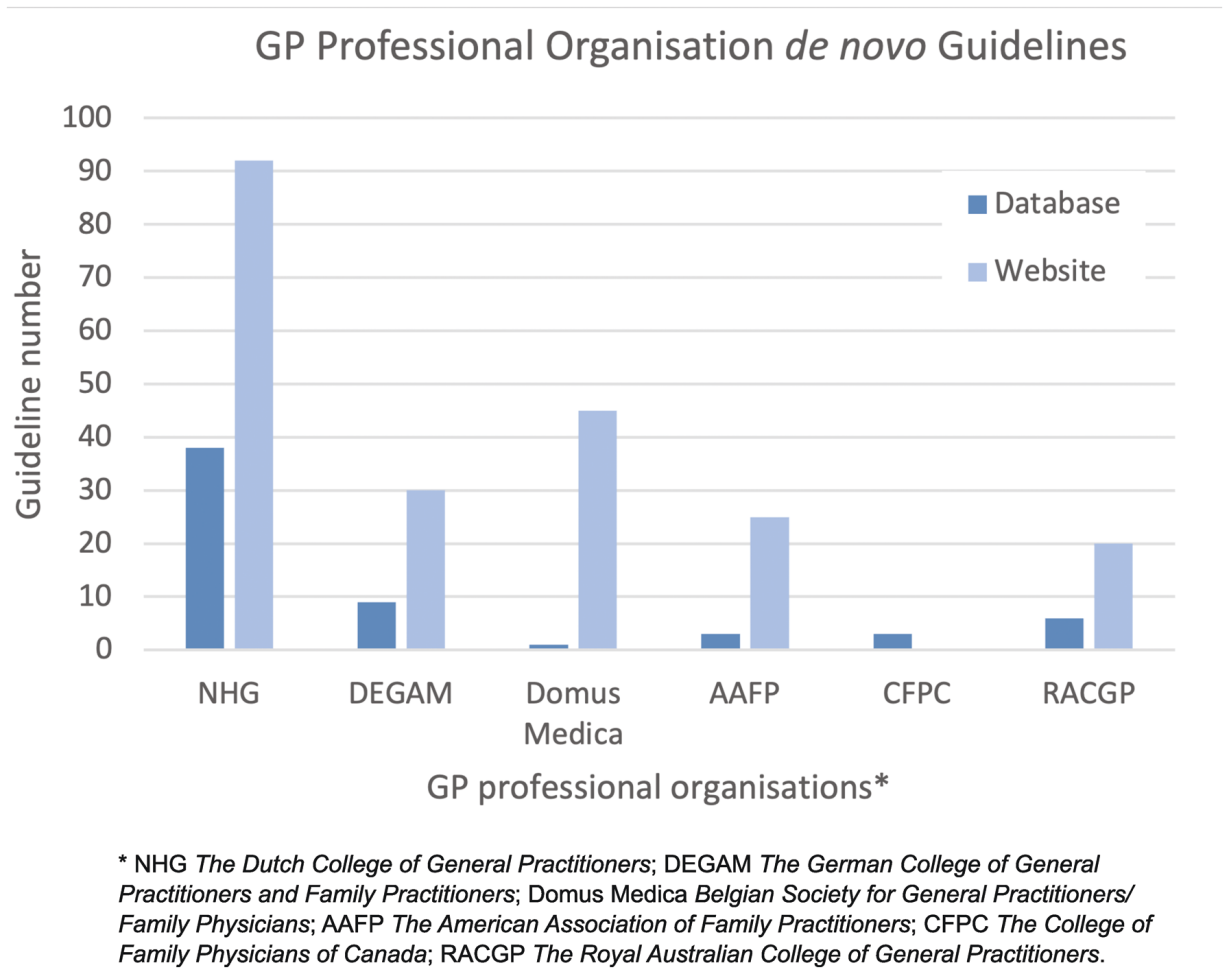
Number of de novo guidelines retrieved from the database search for each general practice professional organization. The number of de novo guidelines found on each organizations website shown for comparison.

The 6 organizations included were from the Netherlands (The Dutch College of General Practitioners [NHG]), Germany (the German College of General Practitioners and Family Practitioners [DEGAM]), Belgium (Belgian Society for General Practitioners/Family Physicians [Domus Medica]), the United States (the American Association of Family Practitioners [AAFP]), Canada (the College of Family Physicians of Canada [CFPC]), and Australia (the Royal Australian College of GPs [RACGP]). From these organizations, 60 guidelines were included, 12 of which were in English and all others were translated (from German or Dutch to English) or accessed in English from the organizations website. Figure 2 shows the number of guidelines retrieved from the search for each organization, the majority were from NHG (*n* = 38).

### Guideline topics

Across the 6 organizations and 60 included guidelines, topics were distributed across 19 clinical categories (Table 3). Mental health comprised the most guidelines (n = 7), addressing broad categories such as anxiety,^32^ depression,^33^ sleep problems,^34^ and addiction management.^35,36^ Two organizations covered very specific guidelines such as managing medically unexplained physical symptoms and somatoform disorder^37^ and depression following coronary syndrome.^38^ In the cardiovascular disease category, chest pain,^39^ atrial fibrillation,^40,41^ venous leg ulcers,^42^ and venous thromboembolism^43^ were identified. Atrial fibrillation is one of only 3 de novo guidelines retrieved from the AAFP^41^; other AAFP guidelines such as cholesterol management and management of hypertension are produced in collaboration with other specialties or by endorsing external organizations such as the US preventive services task force. Chronic disease management, pain management, and preventive care were the top 3 categories (all methods of production) identified from GP organizations (Appendix 4). All organizations identified in the scoping review produce guidelines by collaboration with external organizations and the RACGP, AAFP, and CFPC all endorse guidelines developed by external organizations.

**Table 3.** Summary of guidelines per clinical category.

Mental health (*n* = 7)	Problem drinking^35^	Anxiety^32^	Depression^33^	Medically unexplained physical symptoms^37^	Sleep problems^34^	Depression post coronary syndrome^38^	Opioid use disorder^36^
Cardiovascular disease (*n* = 6)	Atrial fibrillation^40,41,44^	Chest pain^39^	Venous leg ulcers^42^	Venous thromboembolism^43^			
Neurology (*n* = 5)	Facial paralysis^45^	Head injury^46^	Stroke^47,48^	Acute dizziness^49^			
Pregnancy and women’s health (*n* = 5)	Preconception care^50^	Contraception^51^	Menopause^52^	Vaginal bleeding^53^	Labour and delivery after CS^54^		
Preventive care (*n* = 5)	Cardiovascular prevention^55^	Lipid guidelines^56^	Risk prevention (SNAP)^57^	Smoking cessation^58^	Preventive activities in GP^59^		
Sexual health and GU (*n* = 4)	STD consultation^60^	Female urinary incontinence^61^	Sexual problems^62^	Male micturition problems^63^			
Paediatrics (*n* = 4)	ADHD^64^	Obesity^65^	Prolonged cough^66^	Asthma^67^			
Renal and GIT (*n* = 4)	Acute diarrhoea^68^	Gastric symptoms^69^	Diverticulitis^70^	Chronic kidney disease^71^			
Musculoskeletal (*n* = 3)	Lumbosacral radicular syndrome^72^	Hand and wrist symptoms^73^	Osteoarthritis^74^				
Care of the elderly(*n* = 3)	Delirium^75^	Dementia^76,77^					
Respiratory (*n* = 3)	COPD^78^	Acute cough^79^	Asthma^80^				
Endocrine (*n* = 2)	Thyroid disorders^81,82^						
ENT (*n* = 2)	Sore throat^83,84^						
Rheumatology and allergy (*n* = 2)	Food allergy^85^	Polymyalgia rheumatic^86^					
Dermatology and immunology *(n = 1)*	Eczema^87^						
Infectious diseases (*n* = 1)	Influenza pandemic^88^						
Ophthalmology (*n* = 1)	Visual symptoms^89^						
Cannabinoids	^90^						
Genomics	^91^						

### Methods of development of de novo guidelines

All 60 included guidelines contained explicit descriptions of the evidence-based methods involved in their development. A total of 57 guidelines from the 6 organizations followed a similar process of development, outlined in Table 4. In summary, this process involves topic identification and formation of a working group comprising of GPs and other medical specialists, allied health professionals, scientific experts, and patients as appropriate. Once conflicts of interest are declared, guideline-specific questions are formulated. A systematic literature search is conducted, by either an evidence team or member of the working group, depending on the organization and funds available. For example, the RACGP utilize the PEER team while the NHG utilize a methodologist employed by the college. Following the search, recommendations are developed using an approach such as GRADE, utilizing the evidence synthesized,^92^ however use of the GRADE process may depend on external funding.^74^ The final steps of consensus, review and guideline publication is carried out by all 6 organizations.

**Table 4.** Summary of methods of guideline development and updating.

Methods	Topic identification	Working group and conflict of interest develop initial draft	Systematic search	Recommendation (level of evidence and strength of recommendations)	Consensus and draft formation	External review	Final draft	Publication	Citations	Update	Update citations
Dutch College of General Practitioners	✓	✓	✓	✓	✓	✓	✓	✓	^37,46,50,64,70,73^	5–10 years/evidence	^32–35,40,42–45,48,51–53,60–63,65,67–69,72,75,77,78,80,82,84–87,89^
College of Family Physicians of Canada	✓	✓	✓	✓	✓	✓	✓	✓	^36,56,90^	6 years/evidence	
German College of General Practitioners and Family Physicians	✓	✓	✓	✓	✓	✓	✓	✓	^39,49,55,71,76,81,83^		^47,79^
Domus Medica (Belgian College)	✓	✓	✓	✓	✓		✓	✓		5 years	^66^
American Academy of Family Physicians	✓	✓	✓	✓	✓	✓	✓	✓		5 years/evidence	^47,55,71^
Royal Australian College of General Practitioners	✓	✓	✓	✓	✓	✓	✓	✓	^91^	3–5 years	^57–59,74,88^

There was 1 guide from the RACGP^91^ which was distinguished from a guideline, as there was no formulation of weighted recommendations. This was explicitly stated as part of the method of production. Two guidelines were developed using the ADAPTE process,^66,93^ examining currently available international and/or national guidelines as a basis for recommendations, with the remainder of the process following the organizations de novo production method.

Guideline updating processes varied across the 6 organizations. Five of the organizations updated guidelines after a predefined time period, ranging from 3–5 years (RACGP, AAFP, and Domus Medica) to 5–10 years (NHG and the CFPC). The guidelines and web resources from DEGAM did not contain this information.^39,47,49,55,71,76,79,81,83^ In addition to the timeframes specified, the 5 organizations state that guideline updating can be prompted by a change in the evidence and/or national guideline updating on a particular topic. The method is a modified version of the original evidence-based approach (Table 4).

### Structure and dissemination of guidelines

All 6 organizations make guidelines available in downloadable portable document format (Pdf) directly from their organizations website. The length of guideline Pdf’s varied from 30 to 150 pages depending on the topic. Five organizations also provided a summary version of the guideline to accompany the Pdf. DEGAM, NHG, and CFPC provide a summary infographic.^39,47,49,55,71,76,79,81,83^ Websites of NHG, RACGP, and AAFP provide summary versions, but the full version can only be downloaded as a Pdf. Five organizations provide patient information material. For Modus Medica, only the guideline was available via open access, all other material was accessible only to members. Patient information leaflets differ between organizations, e.g. NHG support a dedicated website for patients (https://www.gpinfo.nl/), while the CFPC refers to patient information within their guidelines and also signposts to external patient support groups.

In terms of dissemination, in addition to publishing the full guideline on the organizations website, 3 organizations (CFPC, AAFP, and DEGAM) publish full guidelines, and 1 (NHG) publishes a summary, in their organizations journal. The included Belgium guideline was published in a peer reviewed journal.^66^ Continuing medical education meetings are used for dissemination by CFPC, AAP, NHG, and RACGP. The NHG, RACGP, and CFPC use webinars and e-learning modules, while the NHG and RACGP use periodicals and workshops (Table 5). In the DEGAM guideline on Dementia the strategy for dissemination is described and includes GPs involved in developing the guideline and the publication media drive on completion of a guideline.^76^ Although beyond the scope of this review, of note, clinical decision support systems are used by the CFPC and NHG as a form of implementation, while NHG also use financial incentives and continuing medical education credits.

**Table 5.** Summary of guideline structures and modes of dissemination.

Structure	Pdf	Summary	Patient information leaflet	Education	Audit	Infographics	Citations	Podcast	E-learning module	Periodicals	CME meeting	Faculty study days	Webinars	Workshops	Social media	Newsletter	Peer review publication
NHG	✓	✓	✓	✓	✓	✓	^32–35,37,40,42–46,48,50–53,60–65,67–70,72,75,77,78,80,82,84–87,89,94^		✓	✓	✓	✓	✓	✓	✓	✓	✓
CFPC	✓	✓	✓	✓	✗	✓	^36,56,95^	✓	✓		✓		✓				✓
DEGAM	✓	✓	✓	✓		✓	^39,47,49,55,71,76,79,81,83^										✓
Domus Medica	✓						^66^										✓
AAFP	✓	✓	✗	✗	✗	✗	^38,41,54^				✓				✓	✓	✓
RACGP	✓	✓	✗	✗	✗	✗	^57–59,74,88,91^		✓	✓	✓		✓	✓			✓

## Discussion

### Summary of principal findings

This scoping review included 6 GP professional organizations that publish a range of guidelines for frontline GPs with explicit methods of development and recommendations. Topics cover a wide range of clinical areas including mental health, cardiovascular care, neurology, pregnancy and women’s health and preventive care. These guidelines are available on organizations websites as downloadable Pdfs, with summary documents and patient information. Dissemination strategies include peer reviewed publications, webinars, and continuing medical education meetings.

### Comparison with previous literature

To our knowledge, this review is the first to map guidelines published by GP professional organizations internationally to support GPs in their clinical decision making. Mental health topics were the top clinical category for de novo guideline production. Mental health conditions are a common presenting condition to general practice^96^ and a more significant challenge now due to the COVID-19 pandemic.^97^ Mental health is a leading cause of significant morbidity^98^ and clinical importance is one of the criteria supporting the need for guideline development.^99^ While, GPs dealing with mental health issues may require access to psychologists and psychiatrists,^100^ they remain the first point of care and need diagnostic and therapeutic tools to deal with such presentations. In this context, the updated NHG guidelines on Depression, e.g. now includes these tools as well as website links to online learning modules for GP members to upskill in the delivery of such care.^33^ Chronic health conditions were the main clinical category from GP organizations and topics such as COPD,^78^ asthma,^80^ depression,^33^ obesity,^65^ and chronic kidney disease^71^ were all included guidelines from the review. The complexity of this care is a considerable challenge for frontline GPs, especially as these patients may suffer from multiple chronic conditions.^14^ GPs play a central role in the coordination of this care and for this GP-specific guidance is required.^15^ DEGAM and RACGP publish multimorbidity guidelines on their websites. In addition to guidelines, the literature emphasizes the importance of GPs unique relationships with their patients, the need for policies and “models of practice” that allow nurturing of this relationship in order to manage such complex care.^101^

The findings of this review are consistent with existing literature on guideline development, structure, and updating.^99,102,103^ Each organization has a transparent production method and the de novo process aligns with the Institute of Medicine standards for the development of trustworthy guidelines and the Guideline International Network key components for guideline development.^104,105^ Governance structures and access to government and other national guideline producing bodies,^19^ as well as resources, a need for information sharing and a drive to reduce duplication of effort, all influence the choice of the guideline development process.^106^ The review findings reflect this, as in addition to producing de novo guidelines, all included organizations, produce guidelines by collaborating with other specialities and 50% endorse guidelines from other non GP guideline producing organizations. Guidelines are also developed by adaptation of other available guidelines,^107^ 2 such guidelines are included in this review.^59,66^ The review findings also support other recent literature from Belgium on the quality of evidence-based medicine resources in primary care,^108^ which suggests that being concise, of direct clinical relevance and adapted to the local situation is essential for evidence-based medicine resources.^108^

Recent literature on guideline dissemination focussing on the format and language of guidelines outlines the importance of document structure in helping improve their use in practice. Being user friendly, especially relating to the length of the document and how information is visualized is important for end-users.^109^ Wide variation in the length of guideline pdf documents is evident in this review. Although summary pdfs and summarized website versions of guidelines are available, quick access to pertinent information such as using visual aids and hyperlinks may help to improve their implementation in practice.^110^ The findings of the review show that multiple dissemination strategies are used, including education tools, infographics, patient information, and a publicity drive at the time of guideline publication. The importance of these strategies is highlighted by the findings of a recent Cochrane review of tools to promote uptake of guidelines, where provision of education materials likely improves adherence to guidelines.^111^

### Strengths and limitations

The challenge in this review was to map the landscape of information being produced given the fact that not all GP professional organizations publish their guidelines in peer reviewed journals and also the heterogeneity of the nomenclature for this search. Contacting GP organizations and conducting an extensive grey literature search which included website searches of key organizations helped address this. However, completing a more exhaustive website search (including those organizations whose guidelines were not freely available) and mapping a complete topic list of de novo guidelines from each organization would allow a more detailed comparison of specific guideline recommendations, although that was outside the scope of this review.

This scoping review was limited to de novo production of guidelines, this excluded certain national GP organizations that develop guidelines in association with national bodies. This decision was based on the varying number of GPs that are members of these national bodies and if those GP members represent their organization. Furthermore, in choosing to map the de novo production of guidelines, the full breadth of activity undertaken by GP organizations in terms of guidelines for use in clinical practice was not captured in this review. A good example of this are the AAFP guidelines on Cholesterol Management and Management of Hypertension, both excluded but very relevant for use in GP. The authors believe it is important to map what guidelines GP professional organizations are producing, given the specific challenges for GPs identifying guidelines that are relevant to general practice, as most are single disease focussed, may not account for the patient perspective, and may not consider the challenges relating to cost and resources for general practice.^112^

As this was a scoping review, we did not quality appraise included guidelines so we were not in a position to comment on the overall quality of the included guidelines.

### Research implications

There is scope for further research to map guidelines being published by GP organizations worldwide regardless of the production method. This would facilitate a broader understanding of the complex nature of guidelines necessary for GPs working on the frontline in any given healthcare system.

Implementation strategies were beyond the scope of this review but we did note that in general these were not identified as part of the guidelines identified. There is a gap in the evidence on which type of implementation strategies are effective,^113–115^ and although there is some literature in general practice on these strategies,^116^ there is a need for further research on the type and effectiveness of implementation strategies, e.g. use of financial or other incentives to encourage uptake. International GP organizations would be well placed to collaborate on such research.

### Clinical and policy implications

This review shows that international GP organizations, despite working in different healthcare systems, with different governance structures, produce guidelines with similar topics, methods, and dissemination strategies for use by their members. The standard transparent approach to production methods can facilitate a standardization across GP organizations thus promoting sharing of resources, but this needs to be balanced with the need for local relevance to improve the success of guideline implementation.^117^ Emerging evidence on the effectiveness of guideline implementation in terms of improving patient outcomes, may influence policy around the use of alternative strategies to support the use of guidelines in practice.

## Conclusion

This scoping review has highlighted specific de novo guideline production in GP professional organizations worldwide. There is substantial overlap in the areas of methods of production and publication and some variation in clinical topics and dissemination methods. Overall it indicates there is potential for collaboration between GP organizations worldwide to reduce duplication of effort, facilitate reproducibility, and identify areas of standardization internationally. Furthermore, there is an opportunity for leading guideline producing GP organizations to collaborate with countries where the focus remains on secondary/specialist care, helping to strengthen primary care in these countries.

## Supplementary Material

cmad015_suppl_Supplementary_Checklist

## Data Availability

The datasets generated and/or analysed during the current study are available in the Open Science Framework repository; https://osf.io/cedup/?view_only=12e4fe042a2443dea7345e193a4f5c4d.

## Appendix 1. Search strategy

### Bibliographic database search

**Table:**

	Ovid MEDLINE(R) and Epub Ahead of Print, In-Process, In-Data-Review & Other Non-Indexed Citations, Daily and Versions(R) 1946 to 12 April 2021	
1	general practice.mp. or exp Family Practice/ or exp General Practice/	97421
2	(general ADJ1 practitioner) OR (general ADJ1 practitioners) OR (family ADJ1 practi?e) OR (family ADJ1 physician*)	127219
3	primary health care.mp. or exp Primary Health Care/	182036
4	1 OR 2 OR 3	311989
5	exp Practice Guideline/OR exp Practice Guidelines as Topic/ OR (practice ADJ2 guideline$)	166940
6	((quick adj2 reference adj2 guide*) or (quick adj2 reference) or (evidence adj1 reference) or (evidence adj1 guide*) or (“evidence based” adj1 reference) or (“evidence based” adj1 guide)).mp.	1535
7	5 OR 6	168082
8	4 AND 7	12225
9	LIMIT 8 TO 2010–2021	6214
	EMBASE	
1	“general practice”/exp OR (general NEXT/1 practice) OR (family NEXT/1 practice) OR (general NEXT/1 practioner$)	165374
2	“primary medical care”/exp OR (primary NEXT/2 care)	334254
3	1 OR 2	453204
4	“practice guideline”/mj OR (practice NEXT/1 guideline$)	465331
5	(quick NEXT/1 reference) OR (evidence NEXT/2 reference) OR (evidence NEXT/2 guide*	18087
6	4 OR 5	475402
7	3 AND 6	28513
8	LIMIT 7 TO 2010–2021	17207
9	LIMIT 8 TO EMBASE ONLY RECORDS, EXCLUDING MEDLINE	5285
	COCHRANE LIBRARY	
1	(general NEAR/1 practice) OR (primary NEAR/2 care)	32360
2	practice NEAR/1 guidelin*	13412
3	1 AND 2	1664
4	LIMIT 3 TO REVIEWS OR PROTOCOLS, 2010–2021	260
	WEB OF SCIENCE, Science and Social Citation Indexes LIMITED 2010–2021	
1	TS = (general NEAR/1 practice OR primary NEAR/2 care)	110331
2	TS = (practice NEAR/1 guideline*)	31599
3	1 AND 2	1826

### Grey literature search

**Table:**

*Evidence synthesis* Limited 2010–2021 1396 results for (*“general practice” or “family practice” or “general practitioner” or “family practitioner” or “primary healthcare”) and (“practice guidelines” or “evidence based practice”*) 17 duplicates removed Total 1379
*Guideline central* Search by Speciality, Family Medicine = 851 Filter by Organisation = American Academy of Family Medicine = 4

## Appendix 2. GP organizations questions for supplementary information

**Table:**

1	In what country do you work?
2	What general practice/family physician professional organization do you represent? (e.g. Royal College of General Practitioners, Irish College of General Practitioners, The Royal Australian College of General Practitioners, The Dutch College of General Practitioners)
3	Is this general practice/family physician professional organisation a national organisation? Y/N
4	For this scoping review doctors who work in general practice are defined as: “physicians who provide the first point of contact to patients in the community.” They are available for patients of all ages, with all conditions and provide continuity of care. They are primarily responsible for the “provision of comprehensive and continuing care to every individual seeking medical care irrespective of age, sex and illness” (WONCA 2015). Does this definition represent your general practice/family physician professional organisation’s member’s practice of medicine? Y/N
5	*If you answered “No” to Q4, please provide a brief description of how it differs, otherwise write the word “not applicable” in the box to move to the next question.*
6	Gatekeeping is a core function of general practice in some health systems defined as “matching patient’s needs and preferences with the judicious use of medical services.”^3^ As part of this role, general practitioners authorize access to speciality care, hospital care, and some diagnostic tests. Do the general practice/family physician members of your prof organisation have a gate-keeping role in their practice of medicine?
7	*If you answered “No” to Q6, please provide a brief description of how it differs, otherwise write the word “not applicable” in the box to move to the next question.*
8	Does your general practice/family physician professional organisation produce denovo (new) clinical practice guidelines/guidance documents? Y/N
9	*If you answered “Yes” to Q8, please provide brief details of the process (level of evidence, development team, etc.) involved in the production of these guidelines/guidance documents, otherwise write the word “not applicable” in the box to move to the next question.*
10	How often does your general practice/family physician professional organisation update existing guidelines/guidance documents?
11	Please provide brief details of the process involved in updating existing guidelines, otherwise write the word “not applicable” in the box to move to the next question.
12	Does your general practice/family physician professional organisation adapt existing international guidelines for use within the context of general practice in your healthcare system?
13	Does the general practice/family physician professional organisation endorse national/international guidelines by providing links on the website to these guidelines?
14	*If you answered “Yes” to Q13, what guidelines does your general practice/family physician professional organisation endorse? Please choose as many answers as applicable, or “not applicable” to move to the next question.*
15	Does your general practice/family physician professional organisation allow public/open access to guidelines/guidance documents on their website?
16	If a guideline/guidance document is published in the journal of the GP/FP professional organisation, is this considered a publication of the professional organisation? Y/N
17	We are seeking a list of clinical guideline/guidance categories (regardless of the production process) available to general practitioners/family physicians in your organisation. Please select from the list below or include other categories if not provided in the list Addiction Management Cancer Care Care of the older person Chronic Diseases Domestic Violence Laboratory Investigations Medication review Mental Health Musculoskeletal Medicine Neurology Pandemic and Immunisations Pain Management Paediatric Health Preventive Health Sexual Health Women’s Health Other
18	For the majority of clinical guidelines/guidance documents produced what additional material is available? Please tick as many answers as applicable. Summary document Patient information leaflet Education/Teaching material Audit ideas Infographics Other
19	What are the formats and modes of dissemination of the clinical practice guidelines/guidance documents? Please tick as many answers as applicable. Portable document format (Pdf) Podcast Peer reviewed publications eLearning modules Periodicals Continued medical education meetings Faculty study days Webinars Workshops Other
20	What methods are used for implementation of the guidelines/guidance documents by your general practice/family physician professional organisation? Please tick as many answers as applicable. Clinical decision support system Financial incentives Other incentives Other

## Appendix 3. Covidence database and grey literature search charted guidelines

https://osf.io/cedup/?view_only=12e4fe042a2443dea7345e193a4f5c4d

## Appendix 4. Key Informant Contact responses

https://osf.io/cedup/?view_only=12e4fe042a2443dea7345e193a4f5c4d
